# Anthocyanin-Rich Purple Corn Extract Inhibit Diabetes-Associated Glomerular Angiogenesis

**DOI:** 10.1371/journal.pone.0079823

**Published:** 2013-11-20

**Authors:** Min-Kyung Kang, Soon Sung Lim, Jae-Yong Lee, Kyung Mok Yeo, Young-Hee Kang

**Affiliations:** 1 Department of Food and Nutrition and Center for Aging and Healthcare, Hallym University, Chuncheon, Korea; 2 Department of Biochemistry, School of Medicine, Hallym University, Chuncheon, Korea; 3 S&D Co., Ltd, Chuncheon, Korea; UCL Institute of Child Health, United Kingdom

## Abstract

Diabetic nephropathy (DN) is one of the major diabetic complications and the leading cause of end-stage renal disease. Abnormal angiogenesis results in new vessels that are often immature and play a pathological role in DN, contributing to renal fibrosis and disrupting glomerular failure. Purple corn has been utilized as a daily food and exerts disease-preventive activities. This study was designed to investigate whether anthocyanin-rich purple corn extract (PCE) prevented glomerular angiogenesis under hyperglycemic conditions. Human endothelial cells were cultured in conditioned media of mesangial cells exposed to 33 mM high glucose (HG-HRMC-CM). PCE decreased endothelial expression of vascular endothelial growth factor (VEGF) and hypoxia inducible factor (HIF)-1α induced by HG-HRMC-CM. Additionally, PCE attenuated the induction of the endothelial marker of platelet endothelial cell adhesion molecule (PECAM)-1 and integrin β3 enhanced in HG-HRMC-CM. Endothelial tube formation promoted by HG-HRMC-CM was disrupted in the presence of PCE. In the *in vivo* study employing db/db mice treated with 10 mg/kg PCE for 8 weeks, PCE alleviated glomerular angiogenesis of diabetic kidneys by attenuating the induction of VEGF and HIF-1α. Oral administration of PCE retarded the endothelial proliferation in db/db mouse kidneys, evidenced by its inhibition of the induction of vascular endothelium-cadherin, PECAM-1 and Ki-67. PCE diminished the mesangial and endothelial induction of angiopoietin (Angpt) proteins under hypeglycemic conditions. The induction and activation of VEGF receptor 2 (VEGFR2) were dampened by treating PCE to db/db mice. These results demonstrate that PCE antagonized glomerular angiogenesis due to chronic hyperglycemia and diabetes through disturbing the Angpt-Tie-2 ligand-receptor system linked to renal VEGFR2 signaling pathway. Therefore, PCE may be a potent therapeutic agent targeting abnormal angiogenesis in DN leading to kidney failure.

## Introduction

The long-term complication of diabetes has been implicated as a major contributor to development and progression of pathologic microvascular changes [1], [2]. Diabetic nephropathy (DN) is one of the major diabetic complications and the leading cause of end-stage kidney failure of which prevalence continues to increase [3], [4]. A major feature of DN is microvasculature injury including glomerular hyperfiltration, renal injury and increased urinary albumin excretion, finally leading to glomerular dysfunction and renal failure [3], [5]. The exact cause of DN is unknown, but hyperglycemia, advanced glycosylation products and activation of cytokines have been postulated as various mechanisms [6]. Hyperglycemia-mediated endothelial injury may predispose to albuminuria in diabetes directly and through a communication with neighboring mesangial cells and podocytes [7]. An understanding of the cellular mechanisms of glomerular anomalies in the DN may lead to effective therapies towards prevention and amelioration of DN.

A broad range of anomalies associated with oxygen disorders such as hypoxia and oxidative stress have been implicated in DN [4]. The hypoxic milieu followed by the microvascular rarefaction results in glomerulosclerosis and tubulointerstitial fibrosis [7]. Increased level of blood glucose is thought to have a structural and physiological effect on microvascular capillaries causing them to be both functionally and anatomically incompetent [8]. There is accumulating evidence revealing that hypoxia-inducible factor (HIF)-1α is a key regulator of renal sclerosis under diabetic conditions [9]. Apparently, high blood glucose induces hypoxia in retinal tissues, thus leading to the production of vascular endothelial growth factor (VEGF) for neovascularization [10]. Neo-angiogenesis of glomerular capillaries may take place in early diabetes, particularly in the experimental episodes. Secondary to the induction of VEGF by hypoxia, angiogenesis can be controlled by angiogenic inducers and inhibitors. However, the loss of capillaries in glomeruli is a key event that correlates closely with declining glomerular filtration rate in DN patients [7], [11].

Angiogenesis is an indispensable and physiological process through which new blood vessels form from pre-existing ones. However, abnormal angiogenesis occurs in several complications and is pivotal for tumor growth and metastasis [10], [12]. A major complication of diabetes is angiopathy characterized by abnormal angiogenesis with immature vessels. Abnormal angiogenesis plays a pathological role in diabetic retinopathy, contributing to both vitreous hemorrhage and fibrosis [10]. In DN a pathological role of angiogenesis similar to that observed in retinopathy remains unclear [3]. Hyperglycemia results in the glomerular damage, neovascularization, matrix deposition, and altered filtration [13], [14]. Factors with proangiogenic capacity are VEGF, basic fibroblast growth factor and angiopoietins (Angpt) are well investigated and established to date [15]. VEGF, a potent stimulators of angiogenesis, promotes endothelial cell proliferation, migration, and tube formation and induce vascular permeability [7], [8]. Angpt1, a major physiological ligand for Tie-2 receptor, is responsible for the vascular maturation by inducing recruitment and stable attachment of pericytes [16]. Angpt2, the natural antagonist of Angpt1, fosters sprouting angiogenesis by loosening the attachment of pericytes in the presence of VEGF [16], [17]. Although these angiogenic factors are difficult to manipulate therapeutically, evidence has led to the development of valid therapeutic strategies targeting angiogenesis in DN.

Purple corn, known as Zea mays L., has been widely utilized as food colorant. Purple corn color rich in anthocyanins and functional phenolics has attenuating effects on hypertension, diabetes and cancer as potential medicinal uses [18], [19]. In our previous studies [20], [21], purple corn anthocyanins retarded diabetes-associated glomerular inflammation and glomerulosclerosis. Purple rice bran extract and anthocyanidins suppress VEGF-induced angiogenesis by inhibiting proliferation and migration via the inhibition of activation of ERK and p38 [22]. Based on the possible anti-angiogenic activity of anthocyanins, this study investigated whether anthocyanins-rich purple corn extract (PCE) inhibited excessive blood vessel formation and endothelial proliferation in the early stage of diabetic kidney glomeruli. This study examined whether PCE suppressed the induction of angiogenic factors of VEGF, HIF-1α, Angpt and Tie-2 in endothelial cells cultured in high glucose-exposed mesangial conditioned media (HG-HRMC-CM) and in db/db mice. VEGF receptor 2 (VEGFR2), platelet endothelial cell adhesion molecule (PECAM-1) and Ki-67 were also determined for the anti-angiogenic activity of PCE. Furthermore, endothelial tube formation and aorta ring assay were performed for the PCE antagonism of diabetes-triggered glomerular angiogenesis.

## Materials and Methods

### Materials

Fetal bovine serum (FBS), trypsin-EDTA, and penicillin–streptomycin were purchased from Lonza (Walkersvillle, MD). Dulbecco’s modified Eagle’s media (DMEM), nutrient mixture F-12 Ham medium, mannitol, and D-glucose were obtained from Sigma-Aldrich Chemical (St. Louis, MO), as were all other reagents, unless specifically stated elsewhere. Antibodies of mouse monoclonal HIF-1α, rabbit polyclonal Ki-67 and rabbit polyclonal vascular endothelium cadherin (VE-cadherin) were supplied by Abcam Biochemicals (Cambridge, UK). Goat polyclonal antibodies of PECAM-1, Angpt1, Angpt2, and Tie-2 and were obtained from Santa Cruz Biotechnology (Santa Cruz, CA). Goat polyclonal human/mouse VEGF antibody was provided by R&D systems (Minneapolis, MN), and antibodies of rabbit polyclonal integrin β3, rabbit monoclonal VEGFR2 and rabbit monoclonal phospho-VEGFR2 purchased from Cell signaling Technology (Beverly, CA). Rabbit polyclonal phospho-Tie-2 antibody was provided by Millipore Corporation (Temecula, CA). Mouse monoclonal β-actin antibody was obtained from Sigma-Aldrich Chemical. Horseradish peroxidase-conjugated goat anti-rabbit IgG, goat anti-mouse and donkey anti-goat IgG were purchased from Jackson ImmumnoReserch Laboratories (West Grove, PA).

### PCE Extraction and Preparation

PCE extraction and preparation were described in previous studies [18], [23]. In brief, purple corn kernel powder was obtained from the Brilliant Project International (Seoul, Korea). The powder was applied to a glass open column (10.0×900 mm I.D.) packed with Diaion HP-20 (Mitsubishi Kasei Company, Tokyo, Japan) and eluted with water for washing of sugar or non-phenolic components. Following extraction with 95% ethanol, the phenolic components were used as PCE for the present study.

### Preparation of Conditioned Media of Mesangial Cells and Endothelial Cell Culture

Human renal MC (HRMC, Sciencell Research Laboratories, Carlsbad, CA) were cultured at 37°C humidified atmosphere of 5% CO_2_ in air. Routine culture of HRMC was performed in DMEM plus F-12 (7∶1) media containing 15% FBS, 2 mM glutamine, 100 U/ml penicillin, 100 µg/ml streptomycin. HRMC in passage of 6–10 were sub-cultured at 90% confluence and used for further experiments. To prepare different conditioned media, cells were incubated in serum-free media with 5.5 mM glucose, 5.5 mM glucose plus 27.5 mM mannitol, or 33 mM glucose for 3 d. Subsequently, each culture medium was collected, centrifuged at 1,500 rpm for 10 min to remove cellular debris, decanted into clean tubes, and each conditioned medium was stored at −20°C.

Human umbilical vein endothelial cells (HUVEC) isolated using collagenase were cultured in 25 mM HEPES-buffered M199 containing 10% FBS, 2 mM glutamine, 0.75 µg/ml human epidermal growth factor, and 75 µg/ml hydrocortisone at 37°C humidified atmosphere of 5% CO_2_ in air. HUVEC with cobblestone morphology were passaged at confluence and used for culture within 10 passages. HUVEC were cultured in respective HRMC conditioned media in the absence and presence of 1–20 µg/ml PCE for different times. There was no cytotoxicity of 1–20 µg/ml PCE in HUVEC and HRMC observed [20], [23].

### *In vivo* Animal Experiments

Adult male db/db mice (C57BLKS/+Lepr^db^ Iar; Jackson Laboratory, CA) and their age-matched non-diabetic db/m littermates (C57BLKS/J; Jackson Laboratory) were used in the present study [21], [23]. Mice were kept on a 12 h light/12 h dark cycle at 23±1°C with 50±10% relative humidity under specific pathogen-free conditions, fed a standard pellet laboratory chow diet (CJ Feed, Korea) and were provided with water *ad libitum* at the animal facility of Hallym University. This study employed 8 week-old db/db mice because they develop diabetes (hyperglycemia) at the age of 7–8 weeks [24]. The mice were allowed to acclimatize for a week before beginning the experiments. Mice were divided into three subgroups (n = 8–10 for each subgroup). The first group of mice was non-diabetic db/m control mice and received drinking water as the PCE vehicle. The other db/db mice were orally administrated drinking water or 10 mg/kg BW PCE daily for 8 weeks.

All experiments were approved by the Committee on Animal Experimentation of Hallym University and performed in compliance with the University’s Guidelines for the Care and Use of Laboratory Animals. No mice were dead and no apparent signs of exhaustion were observed during the experimental period [21]. In our previous study physical and biochemical parameters were tested in db/db mice [21]. The body weight of db/db mice was heavier by 20–60% than that of db/m controls. The water intake of db/db mice gradually increased from the first week of the experimentation, indicative of developing diabetes. In addition, the level of plasma HbA1c, a biomarker of diabetic complications, markedly increased in db/db mice. The blood glucose level of db/db animals was much higher than that of db/m controls, which continuously declined after PCE supplementation. Severe albuminuria was observed in db/db mice with a decreased level of urinary creatinine.

### Western Blot Analysis

Western blot analysis was conducted using whole cell lysates prepared from HUVEC at a density of 3.0×10^5^ cells [25]. Kidney tissue extracts were also prepared from mice that were supplemented with PCE. Whole cell lysates and kidney tissue extracts were prepared in a lysis buffer containing 1 M β-glycerophosphate, 1% β-mercaptoethanol, 0.5 M NaF, 0.1 M Na_3_VO_4_ and protease inhibitor cocktail. Cell lysates and tissue extracts containing equal amounts of proteins were electrophoresed on 6–10% SDS-PAGE and transferred onto a nitrocellulose membrane. Nonspecific binding was blocked with 3% bovine serum albumin for 3 h. The membrane was incubated overnight at 4°C with each primary antibody of target proteins and washed in a TBS-T for 10 min. The membrane was then incubated for 1 h with a secondary antibody of goat anti-rabbit IgG, goat anti-mouse IgG, or donkey anti-goat IgG conjugated to horseradish peroxidase. Each target protein level was determined by using Supersignal West Pico Chemiluminescence detection reagents (Pierce Biotechnology, Rockford, IL) and Immunobilon Western Chemiluminescent Horseradish Peroxidase Substrate (Millipore Corp.,Billerica, MA) and Agfa X-ray film (Agfa-Gevaert, Belgium). Incubation with anti-human or anti-mouse β-actin was also performed for comparative control.

### Enzyme-linked Immunosorbent Assay (ELISA)

Mouse plasma levels of VEGF (R&D System) and thrombospondin-1 (USCN Life Science Inc., Wuhan, China) was determined by using ELISA kits according to the manufacturer’s instructions.

### Immunofluorohistochemistry Staining

For the immunohistochemical analyses, paraffin-embedded integument sections (5 µm thick) were employed. The sections were placed on glass slides, deparaffinated, hydrated with xylene and graded alcohol, and pre-incubated in boiling sodium citrate buffer (10 mM sodium citrate, 0.05% Tween 20, pH 6.0) for the antigen retrieval. A specific primary antibody against VE-cadherin, PECAM-1, Ki-67 or VEGFR2 was incubated overnight with tissue sections. For the VE-cadherin visualization, the sections were incubated for 3 h with FITC-conjugated anti-goat IgG. For the double staining of PECAM-1 and Ki-67, the sections were incubated for 3 h with FITC-conjugated anti-goat IgG for PECAM-1 and Cy3-conjugated anti-goat IgG for Ki-67. VEGFR2 was visualized with 3,3′-diaminobenzidine to produce a brown staining, being counterstained with hematoxylin. The stained tissue sections were examined using an optical Axiomager microscope system (Zeiss, Göttingen, Germany) and five images (x400) were taken for each section. Protein levels of VE-cadherin, Ki-67 and VEGFR2 were quantified by image analysis program of the microscope system.

### Mouse Aortic Ring Assay

The mouse aortic ring assay was used as a model for the *ex-vivo* angiogenesis study. Dorsal aorta from a freshly sacrificed C57BL/6 mouse was taken out in a sterile manner and rinsed in ice-cold PBS. Aorta rings were cut into 1 mm-long pieces using a sterile surgical blade. Each ring was placed in a martrigel-pre-coated 24-well plate and treated with different HRMC conditioned media in the absence and presence of PCE or with 10 ng/ml VEGF. On day 6 the rings were photographed by phase-contrast microscopy and the microvessel outgrowth was quantified.

### Endothelial Tube Formation Assay

This study examined endothelial tube formation using a tubular morphogenesis assay. Matrigel Basement Membrane Matrix (BD Biosciences, Heidelberg, Germany) was employed as a substrate for the *in vitro* study of angiogenesis. Matrigel solution was thawed overnight at 4°C and homogenously mixed in a serum free culture medium (1∶1 dilution). Two hundred microliter matrigel solution was distributed onto each 24-well plate and allowed to solidify for 1 h at 37°C. HUVEC were harvested by trypsinization, and 100,000 cells/well were seeded onto 24-well plate pre-coated with martrigel. Cells were incubated for 8 h at 37°C in respective HRMC conditioned media. The branching points were continuously monitored and the tube formation of cells was photographed in five random fields of view per well using a microscopy with CCD camera (Motic, Wetzlar, Germany).

### Data Analysis

The results are presented as mean ± SEM. Statistical analyses were conducted using the Statistical Analysis statistical software package version 6.12 (SAS Institute, Cary, NC). Significance was determined by one-way ANOVA, followed by Duncan’s multiple-range test for multiple comparisons. Differences were considered significant at P<0.05.

## Results

### Suppressive Effects of PCE on Induction of VEGF and HIF-1α

**Figure 1:**
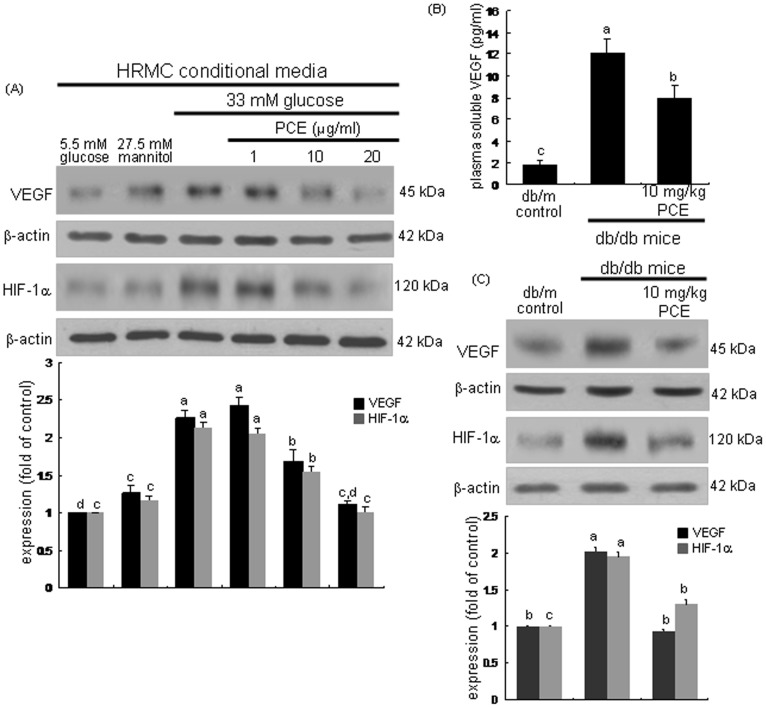
Inhibition of diabetic up-regulation of VEGF and HIF-1α by PCE. HRMC were incubated in 5.5[with (w/) 27.5 mM mannitol and w/33 mM glucose] for 6 h in the absence and presence of 1–20 µg/ml PCE (A). The db/db mice were orally supplemented with 10 mg/kg PCE daily for 8 weeks. The db/m mice were employed as diabetic controls (B and C). Cell lysates and tissue extracts were subjected to Western blot analysis with a primary antibody of VEGF and HIF-1α (A and D). β-Actin protein was used as an internal control. The bar graphs (mean ± SEM, n = 3) in the bottom panels represent quantitative results obtained from a densitometer. Plasma level of VEGF in mice was measured with an ELISA kits (B). Means not sharing a common letter differ, *P*<0.05.

This study examined whether HG-exposed mesangial cells facilitated expressions of proangiogenic factors in glomerular endothelial cells in a paracrine action, which was disrupted by PCE. Endothelial cells were experienced with respective mesangial cell conditioned media collected from HRMC cultured in DMEM/F-12 containing different glucose concentrations (5.5 mM glucose and plus 27.5 mM mannitol or 33 mM glucose). The endothelial expression of VEGF and HIF-1α was enhanced by 6 h-culturing cells in HG (33 mM glucose)-HRMC conditioned media (HG-HRMC-CM, Figure 1A). When 1–20 µg/ml PCE was supplemented to endothelial cells exposed to HG-HRMC-CM, such expression was dose-dependently attenuated. Furthermore, the *in vivo* study attempted to confirm that PCE blocked the induction of the proangiogenic factors of VEGF and HIF-1α in db/db mice. Oral administration of 10 mg/kg PCE reduced the plasma level of VEGF elevated in db/db mice (Figure 1B). In addition, the tissue levels of VEGF and HIF-1α were diminished in PCE-treated mouse kidneys (Figure 1C). Accordingly, hyperglycemia appeared to result in a hypoxic milieu in mouse kidneys and foster neovascular angiogenesis in glomeruli, which was antagonized by treating PCE to diabetic mice.

### Inhibition of Glomerular Cell Proliferation by PCE

**Figure 2:**
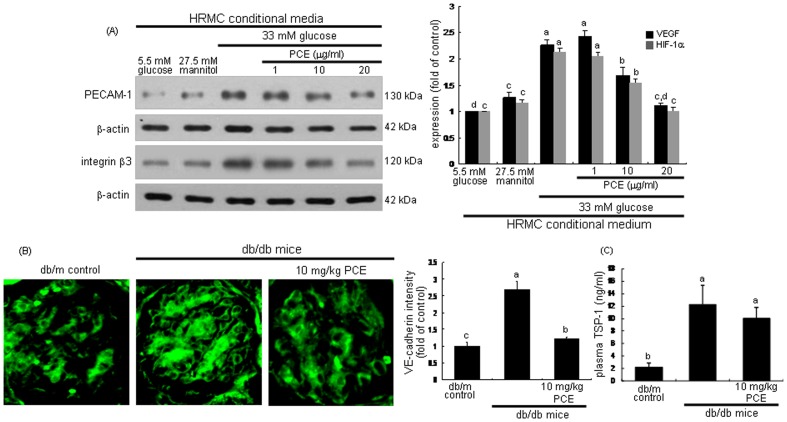
Attenuation of diabetic induction of PECAM-1, integrin β3, VE-cadherin and TSP-1 by PCE. HRMC were incubated in 5.5[with (w/) 27.5 mM mannitol and w/33 mM glucose] for 6 h in the absence and presence of 1–20 µg/ml PCE (A). Cell lysates were subjected to Western blot analysis with a primary antibody of PECAM-1 and integrin β3 (A). β-Actin protein was used as an internal control. The bar graphs (mean ± SEM, n = 5) in the bottom panels represent quantitative results obtained from a densitometer. The db/db mice were orally supplemented with 10 mg/kg PCE daily for 8 weeks (B and C). The db/m mice were employed as diabetic controls. The VE-cadherin induction in histological sections of mouse kidneys was immunohistochemically determined by using anti-mouse VE-cadherin and FITC-conjugated IgG (B). Each photograph is representative of four animals. Magnification: x400. The bar graphs (mean ± SEM, n = 4) in the right panel represent quantitative staining intensity. Plasma level of TSP-1 in mice was measured with an ELISA kit (C). Means not sharing a common letter differ, *P*<0.05.

Angiopathy characterized by abnormal angiogenesis is a major diabetic vascular complication in DN [10]. Abnormal neovascularization is closely linked to glomerular hypertrophy and excess endothelial cell proliferation in DN [6], [7]. The expression of the endothelial adhesion molecules of PECAM-1 and integrin β3 was upregulated in cells cultured in HG-HRMC-CM (Figure 2A). In contrast, PCE deterred the excess induction of endothelial PECAM-1 and integrin β3 by HRMC exposed to HG. PCE may inhibit glomerular hypertrophy due to abnormally enhanced endothelial cell proliferation.

**Figure 3:**
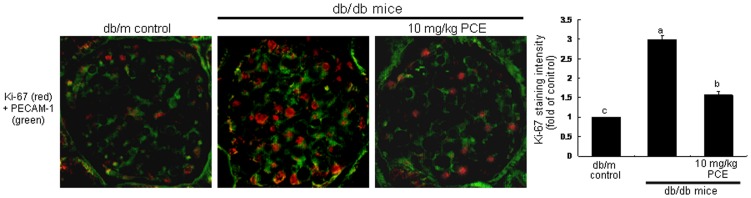
Double-immunofluorescence staining showing PECAM-1 (green) and Ki-67 (red) in db/db mice supplemented with PCE. The db/db mice were orally supplemented with 10 mg/kg PCE daily for 8 weeks. The db/m mice were employed as diabetic controls. For the measurements of the tissue level of Ki-67, histological sections of mouse kidneys were immunohistochemically stained by using anti-mouse Ki-67 and Cy3-conjugated IgG. The Ki-67 level was identified as reddish staining. The sections were also stained with anti-mouse PECAM-1 and FITC-conjugated IgG. Each merged image is representative of four animals. Magnification: x400. The bar graphs (mean ± SEM, n = 3) in the right panel represent quantitative staining intensity of fluorescent Ki-67. Respective values not sharing a letter are different at *P*<0.05.

Immunofluorohistochemical analysis showed that the induction of VE-cadherin was enhanced in glomeruli of db/db mice (Figure 2B). In 10 mg/kg PCE-treated mice, the expression of PECAM-1 decreased compared with that of db/m mice. TSP-1 has been shown to be a natural inhibitor of neovascularization and tumorigenesis in healthy tissue [26]. However, TSP-1 is expressed in newly formed vessels in patients of peripheral arterial disease [27]. The plasma level of the adhesive glycoprotein TSP-1 was elevated in db/db mice, while oral supplementation of 10 mg/kg PCE diminished the elevated plasma level (Figure 2C). In addition, the nuclear protein Ki-67 necessary for cellular proliferation was induced in glomeruli of db/db mice, evidenced by the merged images of double-immunofluorescence staining (reddish staining, Figure 3). However, the PCE treatment significantly suppressed the glomerular Ki-67 induction compared to that of db/m mice.

### Blockade of Microvessel Outgrowth and Tube Formation by PCE

**Figure 4:**
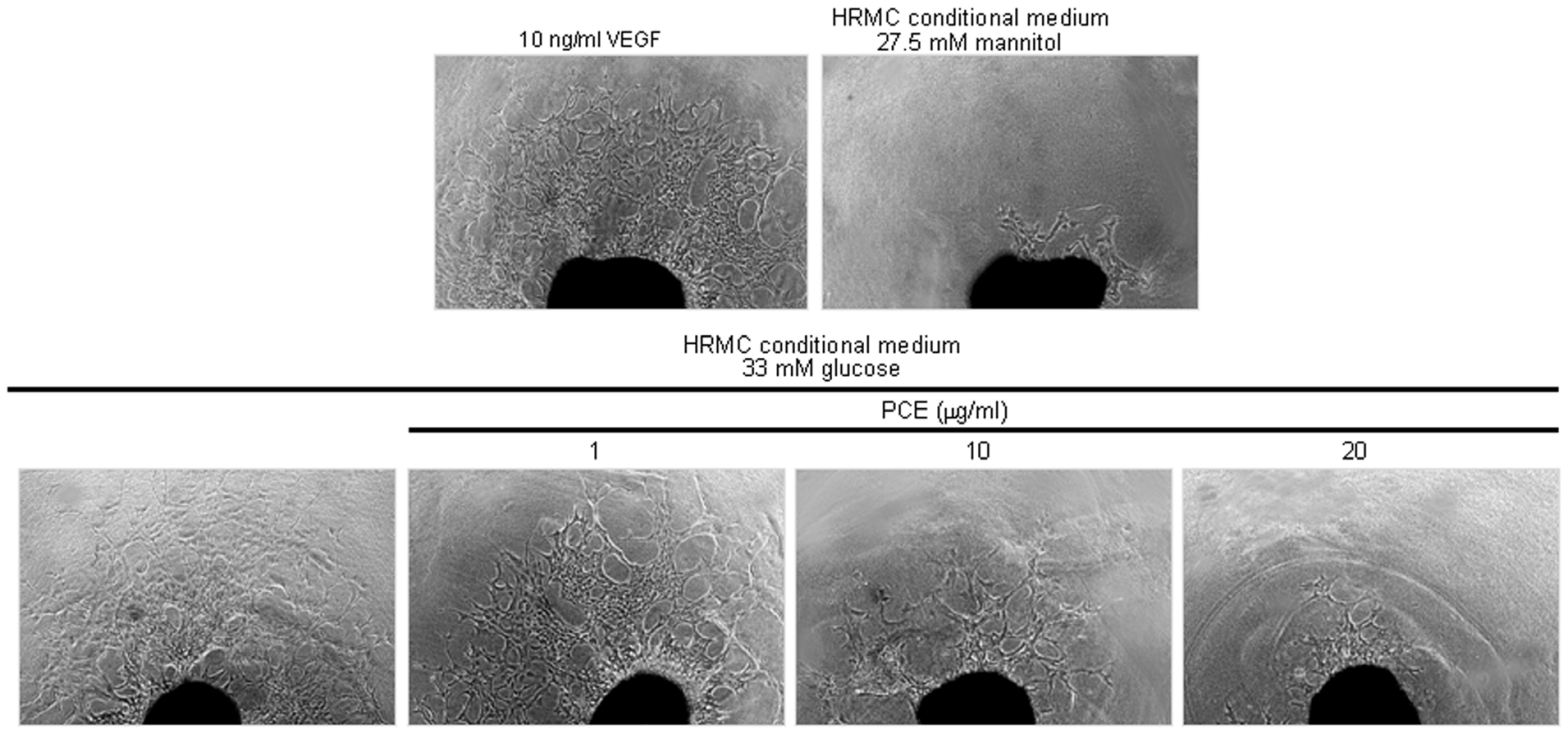
Aortic ring assay showing the diminution of diabetic angiogenesis by PCE. Aorta ring pieces were placed in a martrigel-pre-coated 24-well plate and treated with HRMC conditioned media in the presence of PCE or 10 ng/ml VEGF for 6 d. The microvessel outgrowth from aorta ring pieces was photographed and quantified. Respective values in bar graphs (mean ± SEM, n = 3) not sharing a letter are different at *P*<0.05.

The inhibitory effect of PCE on angiogenesis was assessed by employing an *ex vivo* assay of mouse aortic ring angiogenesis, in which developing microvessels undergo many key features of angiogenesis over a timescale similar to that observed *in vivo*. The treatment of mouse aorta ring with HG-HRMC-CM for 8 d induced microvessel outgrowth similar to that observed with 10 ng/ml VEGF (Figure 4). However, PCE inhibited the microvessel outgrowth in a dose-dependent manner, indicating that PCE can be used as a novel agent interrupting the development of microvessels.

**Figure 5:**
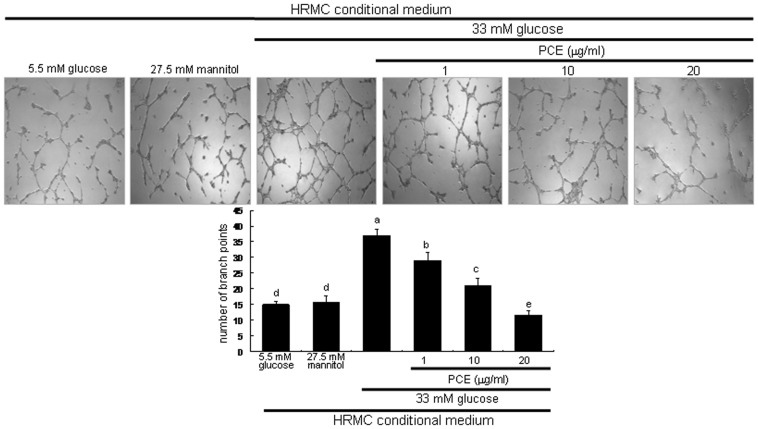
Suppression of endothelial tube formation by PCE. HRMC were incubated in 5.5[with (w/) 27.5 mM mannitol and w/33 mM glucose] for 8 h in the absence and presence of 1–20 µg/ml PCE. Tube formation of HUVEC was assayed using matrigel. Cells were fixed and microphotographic images were captured at ×100 magnification. The branching points were continuously monitored and the number of tubes was counted from the images. Multiple five random fields of view were analyzed for the quantitative results (mean ± SEM, n = 4). Respective values not sharing a letter are different at *P*<0.05.

This study investigated PCE inhibition of tube-like structure formation by using an endothelial tube formation assay on Matrigel designated for the *in vitro* angiogenesis. HUVEC exposed to HG-HRMC-CM for 18 h displayed extensive tubular network with increasing the number of branch points (Figure 5). When 1–20 µg/ml PCE was treated to endothelial cells exposed to HG-HRMC-CM, the tube-like structure formation was dose-dependently suppressed.

### Inhibitory Effect of PCE on Induction of Angpt1, Angpt2 and Tie-2

**Figure 6:**
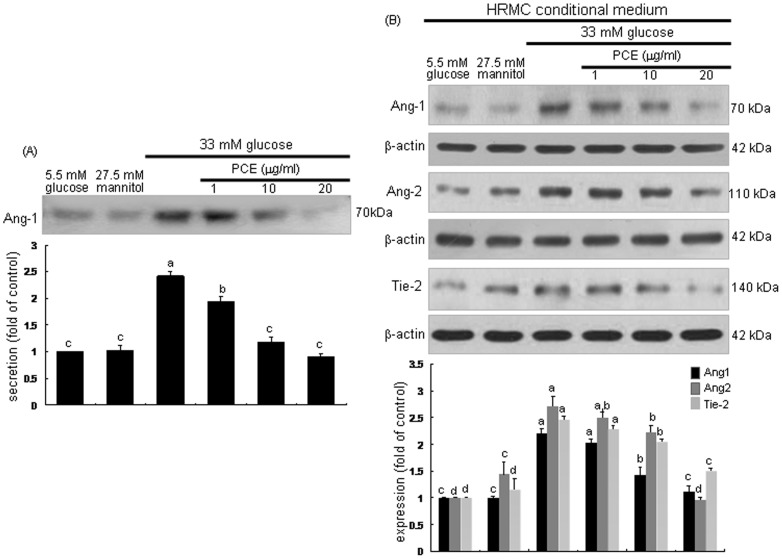
Western blot analysis showing inhibition of diabetic induction of Angpt1, Angpt2 and Tie-2 by PCE. HRMC were incubated in 5.5(A). HUVEC were incubated in HRMC conditioned media [with (w/) 27.5 mM mannitol and w/33 mM glucose] for 6 h in the absence and presence of 1–20 µg/ml PCE (B). Cell lysates were subjected to Western blot analysis with a primary antibody of Angpt1, Angpt2 and Tie-2. β-Actin protein was used as an internal control. The bar graphs (mean ± SEM, n = 3) in the bottom panels represent quantitative results obtained from a densitometer. Respective values not sharing a common letter differ, *P*<0.05.

Angpt1 and Angpt2 are antagonistic ligands that bind to the extracellular domain of Tie-2 receptor almost exclusively expressed by endothelial cells [16], [17]. Angpt1 is constitutively expressed by pericytes and vascular smooth muscle cells [28]. This study examined the Angpt1 secretion of mesangial cells neighboring upon glomerular endothelium. The secretion of mesangial Angpt1 was elevated in HG-HRMC-CM, which was dose-dependently reversed by treating 1–20 µg/ml PCE to mesangial cells (Figure 6A). Endothelial expression of Angpt2 and Tie-2 was markedly promoted by the stimulation with HG-HRMC-CM (Figure 6B). In contrast, the endothelial induction of Angpt2 and Tie-2 was demoted by the presence of 1–20 µg/ml PCE. Additionally, the endothelial Angpt1 induction by HG-HRMC-CM was deterred by the presence of PCE (Figure 6B).

**Figure 7:**
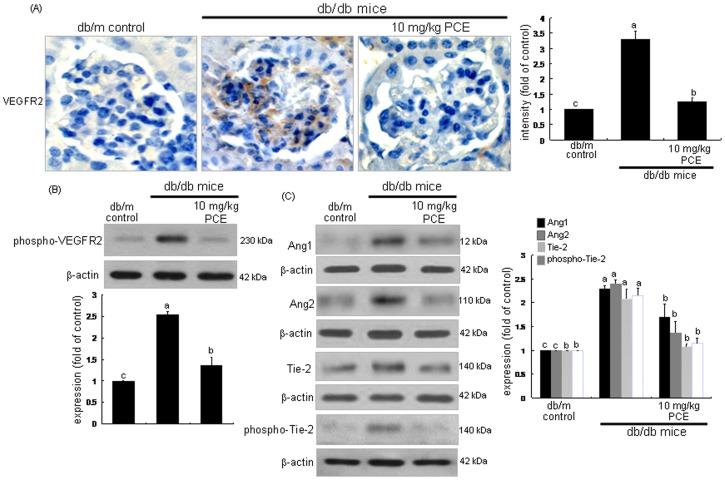
Inhibition of induction of VEGFR2, phospho-VEGFR2, Angpt1, Angpt2, Tie-2 and phospho-Tie-2 in diabetic kidneys by PCE. The db/db mice were orally supplemented with 10 mg/kg PCE daily for 8 weeks. The db/m mice were employed as diabetic controls. For the measurements of the tissue levels of VEGFR2 (A), histological sections of mouse kidneys were immunohistochemically stained by using anti-mouse VEGFR2 and color-fixed with substrate chromogens of 3,3′-diaminobenzidine. The sections were counter-stained with hematoxylin. The VEGFR2 level was identified as brown staining. Each photograph is representative of four animals. Magnification: x400. The bar graphs (mean ± SEM, n = 3) in the right panel represent quantitative staining intensity. For the Western blot analysis (B and C), tissue extracts were subjected to Western blot analysis with a primary antibody of phospho-VEGFR2, Angpt1, Angpt2, Tie-2 and phospho-Tie-2. β-Actin protein was used as an internal control. The bar graphs (mean ± SEM, n = 3) in the bottom panels represent quantitative results obtained from a densitometer. Respective values not sharing a common letter differ, *P*<0.05.

Trafficking and proteolysis of VEGFR2 are required for VEGF-stimulated endothelial signaling and cell migration [29], [30]. Immunohistochemical analysis revealed that the VEGFR2 induction was highly enhanced in glomeruli of db/db mice (brown staining, Figure 7A). Oral supplementation of 10 mg/kg PCE to db/db mice dampened the VEGFR2 induction. Furthermore, the PCE treatment diminished the tissue level of phosphorr-VEGFR2, indicating that PCE blunted the VEGFR2 activation (Figure 7B). On the other hand, Angpt1, Angpt2, Tie-2 and phospho-Tie-2 for the neovascularization from preexisting vessels were examined in db/db mouse kidneys. Tissue levels of Angpt1, Angpt2 and Tie-2 were elevated in db/db mouse kidneys, and such elevation was antagonized by oral administration of 10 mg/kg PCE to db/db mice (Figure 7C). Additionally, the Tie-2 activation was enhanced in db/db mouse kidneys, which was blocked by administrating PCE (Figure 7C). Accordingly, PCE inhibited glomerular angiogenesis by disturbing the cell-cell stabilization of Angpt1 required for the maturation of new blood vessels and its destabilization of Angpt2 involved in the endothelial migration and proliferation in presence of VEGF.

## Discussion

Eight major findings were extracted from this study. 1) PCE inhibited endothelial expression of VEGF and HIF-1α induced by HG-HRMC-CM. 2) Oral administration of PCE dampened renal induction of VEGF and HIF-1α concomitant with the reduction of plasma level of soluble VEGF in diabetic mice. 3) PCE suppressed the marked induction of endothelial proliferation markers of PECAM-1 and integrin β3 in HG-HRMC-CM-exposed endothelial cells. 4) PCE supplementation to diabetic mice inhibited the induction of VE-cadherin and Ki-67 in glomeruli and reduced plasma TSP-1 level. 5) Microvessel outgrowth from mouse aorta rings cultured in HG-HRMC-CM for 8 d was blunted by the PCE presence. 6) In endothelial cells exposed to HG-HRMC-CM with PCE the tube-like structure formation was suppressed. 7) PCE attenuated the proangiogenic induction of Angpt2, Tie-2 and Angpt1 in endothelial cells exposed to HG-HRMC-CM containing mesangial Angpt1. 8) Administration of PCE diminished the tissue levels of Angpt2, Tie-2, Angpt1 and phospho-Tie-2 as well as the induction and activation of VEGFR2 elevated in diabetic kidney. Therefore, PCE may antagonize excessive blood vessel formation of glomeruli instigated due to hyperglycemia-hypoxic milieus in the early stages of diabetes.

Increased levels of blood glucose are thought to result in pathological and structural anomalies in microvascular capillaries and to render them functionally and anatomically incompetent [8]. It was shown that VEGF-A activity increased in the endothelium of mildly injured glomeruli [31]. Upregulation of VEGF-A may be a proangiogenic mechanism for the initial progression for excessive blood vessel formation in early stages of DN [3]. Hyperglycemia induces hypoxia in retinal tissues, thus leading to the production of VEGF for the neovascularization in diabetic retinopathy [10]. In advanced diabetic kidney diseases the hypoxic situation owing to microvascular rarefaction results in glomerulosclerosis and tubulointerstitial fibrosis [7]. Chronic hypoxia and abnormal oxygen metabolism have been implicated in DN [4]. The antagonistic key factor against hypoxia is HIF-1α and it is a key regulator of renal sclerosis under diabetic conditions [9]. An understanding of the cellular mechanisms of glomerular anomalies in DN may lead to effective therapies towards prevention and amelioration of DN. This study examined whether PCE inhibited the enhanced angiogenesis in the early stage of diabetic kidney glomeruli. Endothelial HIF-1α was induced in diabetes-mimic milieus and diabetic kidneys, and the HIF-1α induction was inhibited by the presence of PCE. It should be noted that HUVEC were employed as a model of microvascular endothelial cells within glomeruli to allow *in vitro* assessment of their properties [32]. The induction of endothelial HIF-1α cells was examined in cells cultured in conditioned media of HG-exposed mesangial cells anatomically adjacent to glomerular endothelial cells. Our previous study showed that PCE was antagonistic to diabetes-linked glomerulosclerosis and mesangial fibrosis [21], [23]. Unfortunately, this study did not examine the hypoxic status in HG-HRMC-CM-exposed endothelial cells or in diabetic kidneys. Nevertheless, the blockade of neovasculization by PCE may be attributed to its inhibition of HIF-1α induction caused by diabetes-associated microvascular hypoxia of glomeruli mildly injured in the early stage of DN.

It has been shown that HIF-1 upregulates the expression of adhesion proteins of integrins, soluble growth factors of VEGF and transforming growth factor-β and extracellular matrix components [33], [34]. We did not examine direct reciprocal interaction of HIF-1α and VEGF under diabetic milieus. When endothelial cells were cultured in HG-HRMC-CM, the cellular expression of VEGF, and the adhesion molecules of integrin β3 and PECAM-1 was highly upregulated. In addition, similar upregulation of VE-cadherin was observed in diabetic mouse kidneys. Interestingly, in db/db mice with DN the plasma TSP-1 level was elevated. TSP-1 is a plasmatic marker of peripheral arterial disease that modulates endothelial progenitor cell-induced angiogenesis [27]. PCE suppressed the induction of VEGF, PECAM-1, integrin β3, VE-cadherin and/or Ki-67 in HG-HRMC-CM-exposed endothelial cells and in diabetic kidneys. Accordingly, PCE may be a potent inhibitor of VEGF-induced neovascularization, hyperplasia and hypertrophy in DN. In fact, PCE disturbed endothelial tube formation and microvessel outgrowth of aortic rings. Similarly, purple rice bran extract and anthocyanidins suppress VEGF-induced angiogenesis by inhibiting proliferation and migration via the inhibition of activation of ERK and p38 [22]. Delphinidin, a vasoactive polyphenol belonging to the class of anthocyanin, dampens endothelial cell proliferation and migration as well as *in vivo* angiogenesis [35]. The inhibitory effects of anthocyanidins including delphinidin, cyanidin and malvidin on angiogenesis may be responsible for their antioxidant activity [36]. Hyperglycemia-associated ROS production contributes to the glomerular damage, neovascularization and altered glomerular filtration observed in DN [13]. It can be speculated that PCE may disturb VEGF signaling for neovasculization linked to diabetes-associated microvascular hypoxia.

A major complication of diabetes is angiopathy that is characterized by abnormal angiogenesis, leading to formation of new vessels that are often immature in DN [10]. Thus, the inhibition of angiogenesis ameliorates renal alterations with maintenance of podocyte phenotype in type 2 DN [14]. The current study revealed that PCE was capable of disrupting proangiogenic signaling by blocking the induction and activation of VEGFR2 in diabetic glomeruli. Another class of proangiogenic factors is Angpt proteins that are well investigated and established to date [16]. Recently, targeted Angpt-1 therapy shows promise as a renoprotective tool in the early stages of diabetic kidney disease [37]. PCE attenuated the induction of Angpt1, Angpt2 and Tie-2 in endothelial cells cultured in HG-HRMC-CM and in diabetic kidneys. Accordingly, PCE concurrently antagonized sprouting angiogenesis promoted by Angpt2 and vascular maturation aided by Angpt1. PCE appeared to disrupt the endothelial-specific Angpt-Tie ligand-receptor system, being an antagonistic regulator of endothelial activation. It has been reported that Angpt2 and Tie-1 downregulate Angpt1-induced Tie-2 signaling, and Angpt actions in kidney development are further modified by VEGFA and integrins [38]. It should be noted that glomerular component mesangial cells produced Angpt1, which was thought to induce Tie-2 and Angpt2 in neighboring endothelial cells. Green tea polyphenol epigallocatechin-3-gallate inhibits the invasion and migration of prostate carcinoma LNCaP cells through suppressing the protein expression of VEGF and Angpt [39]. Novel therapeutic approaches that target vascular maturation and normalization are now being developed to protect kidneys from capillary rarefaction and hypoxic injury due to diabetic complications [40]. One report has shown that there are increases in renal expression of Angpt2 as well as VEGFA and transforming growth factor-β1, which is inhibited by a small molecule isocoumarin 2-(8-hydroxy-6-methoxy-1-oxo-1H-2-benzopyran-3-yl) propionic acid with antiangiogenic activity in db/db mice [41]. The Angpt manipulation by PCE has potential therapeutic applications in inhibiting diabetic retinal neovascularisation as well as in promoting glomerular repair.

In summary, the present study revealed that PCE disturbed the induction of VEGF and HIF-1α in endothelial cells incubated in HG-HRMC-CM and in diabetic kidneys. PCE blunted endothelial hyperplasia by attenuating the induction of PECAM-1 and integrin β3 in HG-HRMC-CM-exposed endothelial cells. In the *in vivo* study PCE ameliorated diabetes-associated glomerular hypertrophy by suppressing the renal induction of Ki-67 as well as VE-cadherin in db/db mice. The capability of PCE to deter angiogenesis in diabetic glomeruli may come from disturbing the Angpt-Tie-2 ligand-receptor system linked to renal VEGFR2 signaling pathway. Therefore, the renoprotection of PCE against diabetic neovasculization and glomerular hypertrophy may be a specific therapy targeting diabetes-associated diabetic abnormal angiogenesis. In addition, the PCE supplementation would be an important strategy for preventing renal angiopathy in type 2 diabetes.
